# Recurrence of visceral and muco-cutaneous leishmaniasis in a patient under immunosuppressive therapy

**DOI:** 10.1186/s12879-017-2571-x

**Published:** 2017-07-07

**Authors:** Gilles Darcis, Gert Van der Auwera, Jean-Baptiste Giot, Marie-Pierre Hayette, Françoise Tassin, Jorge Arrese Estrada, Lieselotte Cnops, Michel Moutschen, Laurence de Leval, Philippe Leonard

**Affiliations:** 1 Centre Hospitalier Universitaire (CHU) de Liège, Liège, Belgium; 2 Institute of Tropical Medicine, Antwerp, Belgium; 3 Service of clinical Pathology, Lausanne University Hospital, Lausanne, Switzerland

**Keywords:** Cutaneous leishmaniasis, Mucosal leishmaniasis, Visceral leishmaniasis, Immunosuppression, Parasitology, Microbiology

## Abstract

**Background:**

Leishmaniasis is a protozoan disease caused by parasites of the genus *Leishmania*, transmitted to humans by sandflies. The diagnosis of leishmaniasis is often challenging as it mimics many other infectious or malignant diseases. The disease can present in three ways: cutaneous, mucocutaneous, or visceral leishmaniasis, which rarely occur together or consecutively.

**Case presentation:**

The patient was a 52 years old immunosuppressed Belgian woman with a long history of severe rheumatoid arthritis. She underwent bone marrow biopsy to explore thrombocytopenia. Diagnosis of visceral leishmaniasis was made by identification of Leishman Donovan (LD) bodies in macrophages. Treatment with liposomal amphotericin B was successful. She later developed cutaneous leishmaniasis treated with amphotericin B lipid complex. She next presented with relapsing cutaneous lesions followed by rapidly progressing lymphadenopathies. Biopsy confirmed the diagnosis of leishmaniasis. Treatments by miltefosine, amphotericin B, N-methyl-glucamine antimoniate were subsequently initiated. She later presented a recurrent bone marrow involvement treated with intramuscular paromomycin and miltefosine. She died two years later from leukemia. At the time of death, she presented with a mucosal destruction of the nose. A *Leishmania*-specific PCR (Polymerase Chain Reaction) identified *L. infantum* as etiological agent.

**Conclusions:**

Clinicians should be aware of the potential concomitant or sequential involvement of multiple anatomic localizations of Leishmania in immunosuppressed patients.

## Background

Leishmaniasis is a protozoan disease caused by more than 20 species of the genus *Leishmania* and transmitted to humans by the bite of sandflies. The diagnosis of leishmaniasis is often challenging as it mimics many other infectious or malignant diseases.

Three patterns of infection are described: cutaneous (CL), mucosal (ML) and visceral leishmaniasis (VL). CL usually presents as papules, plaques, ulcers or nodules and occurs mostly on exposed areas of the skin. ML is characterized by destructive lesions mainly affecting the nose and the mouth. VL, also known as kala-azar or black fever, is a potentially fatal disease. Symptoms include fever, weight loss, hepato- and splenomegaly. Anemia and thrombocytopenia occur when parasites accumulate in the bone marrow. VL widely predominates in poor rural and suburban areas of India, Bangladesh, Sudan, Ethiopia and Brazil while CL is more broadly distributed and occurs in the south and central American countries, the Mediterranean basin, and from the Middle East to Central Asia [1]. ML is mostly observed in Latin America.

*L. infantum* is well recognized as the etiological agent of VL in southern Europe and is much less commonly reported as a cause of CL [2]. Mucosal forms of *L. infantum* infection are very rare [3–5]. Concomitant or consecutive cutaneous or mucosal with visceral clinical manifestations of leishmaniasis have been seldom described. Herein we report the first case of an immunosuppressed woman who presented the entire spectrum of the disease and we discuss its clinical significance.

## Case presentation

The patient was a Belgian woman born in 1956 with a long history of severe rheumatoid arthritis (RA) complicated by vasculitis and cryoglobulinemia, and treated with etanercept, ciclosporin and methylprednisolone. She had only travelled to Spain in 2004.

**Fig. 1:**
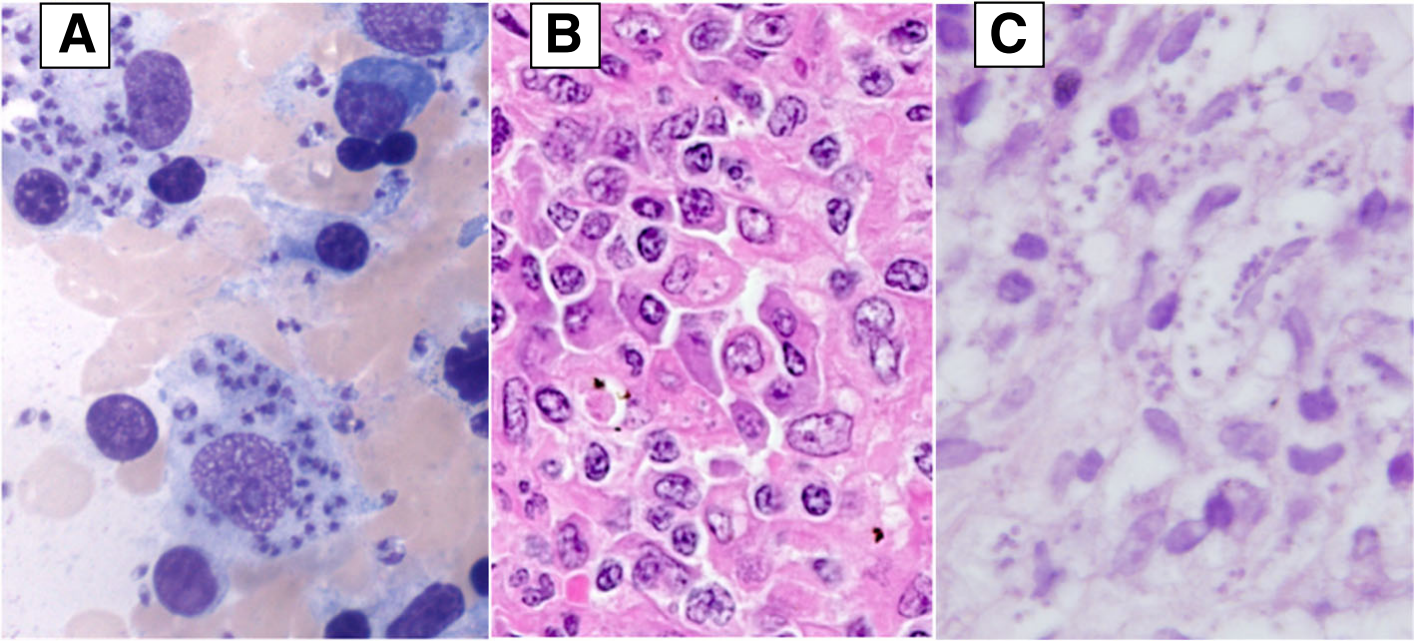
Visceral and cutaneous leishmaniasis. **a**. Bone marrow aspirate: May Grunwald Giemsa (original magnification: ×1000) showing two macrophages with abundant cytoplasm containing innumerable *Leishmania* amastigotes. **b**. Spleen histology: High power view of the splenic red pulp comprising a prominent infiltrate of histiocytes and plasma cells; the histiocytes show numerous cytoplasmic punctate bodies surrounded by a clear halo, suggestive of Leishman bodies (hematoxylin eosin staining; original magnification: ×400). **c**. Cutaneous histology: Diffuse infiltrate in the upper dermis consisting of predominantly large histiocytes, plasma cells and a few lymphocytes. Protozoan *leishmania*, visualized as multiple gray-blue bodies within the vacuolated cytoplasm of histiocytes (hematoxylin eosin staining)

In 2006 she developed mild anemia, thrombocytopenia and splenomegaly which were interpreted as manifestations of Felty syndrome. Screening for infectious diseases including HIV was negative. In March 2007 she was diagnosed with stage IIB EBV-negative classical Hodgkin lymphoma, based on an inguinal lymph node biopsy. The patient was treated with four cycles of C-MOPP (cyclophosphamide, vincristine, procarbazine and prednisone) plus radiation therapy targeting the left inguinal region. While undergoing chemotherapy she had persistent neutropenia (neutrophils: 1390/mm^3^, norm: 2100–8000/mm^3^) thrombocytopenia (thrombocytes: 43,000/mm^3^, norm: 170,000–400,000/mm^3^) and increasing splenomegaly. The markedly enlarged spleen (850 g) was surgically removed and showed marked histiocytic infiltrate of the red pulp, hemophagocytosis, and reactive polytypic plasma cells. Since thrombocytopenia was still increasing, she underwent bone marrow biopsy and aspirate in 2008. Diagnosis of VL was made by identification of Leishman Donovan (LD) bodies in macrophages after Giemsa staining (Fig. 1a). Serum antibody tests (indirect immunofluorescence) were positive for *L. braziliensis*, *L. infantum* and *L. tropica* due to cross reactions between species. In retrospect, *Leishmania* was also detected in the splenic sections (Fig. 1b).

Treatment with liposomal amphotericin B (4 mg/kg for 10 days) was successful. Due to immunosuppression, secondary prophylaxis with monthly amphotericin B was given as proposed for patients with HIV coinfection [6], but stopped after only 2 doses because of medical costs issues.

In 2009, etanercept treatment was replaced by rituximab twice a year. She developed multiple cutaneous nodular lesions mainly located in the left elbow and the buttocks. Histopathology was performed. LD bodies were evident within macrophages (Fig. 1c). Amphotericin B lipid complex treatment (5 mg/kg for 10 days) allowed for complete healing of the lesions. Secondary prophylaxis with monthly amphotericin B was again given. In 2010, despite secondary prophylaxis, she presented with relapsing cutaneous lesions followed by rapidly progressing lymphadenopathies (cervical, subclavicular, sub- and sus-diaphragmatic) on PET/CT. Lymph node biopsy showed massive macrophagic infiltrate comprising LD bodies. Rituximab was stopped. Treatment by miltefosine (50 mg bd) was instituted but then stopped because of digestive intolerance. Amphotericin B was reintroduced but failed, probably because of resistance. N-methyl-glucamine antimoniate (20 mg/kg), more recently used with success by other groups when multiple failures or relapses occur after treatment with liposomal amphotericin B [7], was subsequently initiated and then stopped after the patient experienced torsade de pointes. Cutaneous lesions disappeared and PET/CT showed an almost full metabolic response with disappearance of adenopathy. However, she later presented thrombocytopenia due to recurrent bone marrow involvement which was successfully treated with intramuscular paromomycin (15 mg/kg) and miltefosine (50 mg bd) for 28 days.

**Fig. 2:**
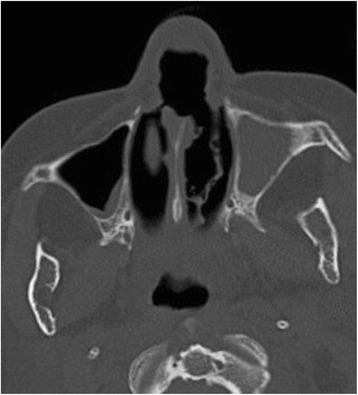
Mucosal leishmaniasis. CT scan showing left maxillary sinusitis and centimetric perforation of the nasal septal cartilage

In 2012, she presented few relapsing cutaneous lesions requiring topical treatment. Two years later she was diagnosed with chronic myelomonocytic leukemia and died in 2015 from blastic crisis. At the time of death, she presented with a mucosal destruction of the nose clearly visible on a CT scan performed a few days before she died (Fig. 2). Autopsy was performed to confirm the mucosal involvement by *Leishmania*. A real-time *Leishmania*-specific PCR [8] performed on the nasal biopsy was positive (cycle threshold value 23,87) and molecular HSP70-typing identified *L. infantum* as etiological agent [9].

## Discussion and conclusion

*L. infantum* is the etiological agent of VL in Europe where it is endemic in the Mediterranean region. Dogs are considered the major reservoir and sandflies are the only proven vectors of this zoonotic protozoan disease [10]. The incidence of VL associated with the transmission of *L. infantum* has been declining in many foci where living standards have improved. Immunosuppression confers a higher risk of clinical disease due to *L. infantum*. While visceral and cutaneous manifestations of *L. infantum* are quite common, mucosal presentation is rare and has been only sporadically described. Association of cutaneous and visceral involvement has been reported in patients co-infected with HIV [11]. Simultaneous mucosal and visceral manifestations have been described in corticosteroid-treated patients [12]. Interestingly, Souza et al. very recently reported patients from an endemic area of leishmaniasis, treated for RA, who presented with recurrent CL or ML [13]. However, we believe that this case report is the first example of *L. infantum* infection with recurrent visceral, cutaneous and finally mucosal involvement. The development of all these clinical manifestations has been facilitated by immunosuppressive therapy required to control a severe RA.

This case also demonstrates that the diagnosis of leishmaniasis is often challenging as it mimics many other infectious or malignant diseases. The scarcity of leishamniasis in non-endemic settings like Belgium makes the diagnosis even more difficult and contributed to the delay before diagnosis. Thrombocytopenia was first thought related to hypersplenism. Following splenectomy, persistent thrombocytopenia could be caused by numerous conditions and manifestations of VL are often misinterpreted as symptoms of malignancies, other infectious or autoimmune diseases such as systemic lupus erythematosus [14]. Discussion of cutaneous lesions is also challenging since CL mimics other infectious conditions including mycobacteriosis or inflammatory diseases such as nummular dermatitis or psoriasis. Moreover, cutaneous lesions could have been considered as post-kala-azar dermal leishmaniasis. However, the recurrence of cutaneous lesions together with VL relapse as well as the localized nature of the cutaneous lesions rather suggests CL. Diagnosis of mucosal lesions located in the nose is complex since lesions often mimic neoplastic processes or necrotizing vasculitis [15].

In conclusion, this exceptional case highlights that clinicians should be aware of the potential concomitant or sequential involvement of multiple anatomic localizations of leishmaniasis in immunosuppressed patients. This observation is particularly relevant given the risk of emergence of leishmaniasis in Europe consequent to the increasing worldwide travelling of humans and domestic dogs, increasing flow of migrants from the Middle East to Europe and climatic changes [16]. Indeed, the occurrence of sandflies in Central Europe and in adjacent regions of Western-Europe is established and sandflies have been found in several parts of Germany and Belgium [17]. Thus local acquisition of leishmaniasis in Belgium is not totally excluded.

## Abbreviations

CL: Cutaneous leishmaniasis
C-MOPP: Cyclophosphamide, vincristine, procarbazine and prednisone
LD bodies: Leishman Donovan bodies
ML: Mucosal Leishmaniasis
PCR: Polymerase Chain Reaction
VL: Visceral leishmaniasis
